# Making change last: applying the NHS institute for innovation and improvement sustainability model to healthcare improvement

**DOI:** 10.1186/1748-5908-8-127

**Published:** 2013-10-26

**Authors:** Cathal Doyle, Cathy Howe, Thomas Woodcock, Rowan Myron, Karen Phekoo, Chris McNicholas, Jessica Saffer, Derek Bell

**Affiliations:** 1 NIHR CLAHRC for Northwest London, 4th Floor Lift Bank D, Chelsea & Westminster Hospital, 369 Fulham Road, London SW10 9NH, UK

**Keywords:** Sustainability, Implementation

## Abstract

The implementation of evidence-based treatments to deliver high-quality care is essential to meet the healthcare demands of aging populations. However, the sustainable application of recommended practice is difficult to achieve and variable outcomes well recognised. The NHS Institute for Innovation and Improvement Sustainability Model (SM) was designed to help healthcare teams recognise determinants of sustainability and take action to embed new practice in routine care. This article describes a formative evaluation of the application of the SM by the National Institute for Health Research Collaboration for Leadership in Applied Health Research and Care for Northwest London (CLAHRC NWL).

Data from project teams’ responses to the SM and formal reviews was used to assess acceptability of the SM and the extent to which it prompted teams to take action. Projects were classified as ‘engaged,’ ‘partially engaged’ and ‘non-engaged.’ Quarterly survey feedback data was used to explore reasons for variation in engagement. Score patterns were compared against formal review data and a ‘diversity of opinion’ measure was derived to assess response variance over time.

Of the 19 teams, six were categorized as ‘engaged,’ six ‘partially engaged,’ and seven as ‘non-engaged.’ Twelve teams found the model acceptable to some extent. Diversity of opinion reduced over time. A minority of teams used the SM consistently to take action to promote sustainability but for the majority SM use was sporadic. Feedback from some team members indicates difficulty in understanding and applying the model and negative views regarding its usefulness.

The SM is an important attempt to enable teams to systematically consider determinants of sustainability, provide timely data to assess progress, and prompt action to create conditions for sustained practice. Tools such as these need to be tested in healthcare settings to assess strengths and weaknesses and findings disseminated to aid development. This study indicates the SM provides a potentially useful approach to measuring teams’ views on the likelihood of sustainability and prompting action. Securing engagement of teams with the SM was challenging and redesign of elements may need to be considered. Capacity building and facilitation appears necessary for teams to effectively deploy the SM.

## Background

The implementation of evidence-based treatments or technological innovations that demonstrate the delivery of high-quality care at acceptable cost is essential if we are to meet the healthcare demands of aging populations [1]. However, evidence suggests efforts to introduce evidence-based new practice frequently fail to apply it in a sustainable way, resulting in variable outcomes [2-4].

The National Health Service (NHS) Institute for Innovation and Improvement Sustainability Model [5] (SM) was designed to help teams implementing new practice in their workplace to address this problem. It aims to enable teams to recognize and self-assess against key variables in their local context that determine whether a new practice is likely to be sustained and to prompt timely action to increase the likelihood of this being achieved. Sustainability in this context means the continuation or the integration of new practice within an organization whereby it has become a routine part of care delivery and continues to deliver desired outcomes [6-8].

The SM is a self-assessment tool that details ten key determinants or ‘key factors’ that increase the likelihood of sustainability and continuous improvement. The model was developed using information gathered from various sources. A review of management literature related to sustainability and research involving project leaders, directors, clinicians, and global health care experts within a national NHS Improvement Program initially identified over 100 factors considered to be important ingredients for sustaining change. Through focus groups and other means, 250 NHS staff and health care experts were asked to rank these factors from 1 to 10 and from this the final 10 factors were derived [5]. Table 1 provides a table listing the ten factors and synopsizes what they are attempting to measure.

**Table 1:** Factors proposed to affect likelihood of sustainability

**Domain**	**Factor**	**Issues being explored**
Process	Factor 1: Benefits beyond helping patient	Whether in addition to helping patients there are other benefits that will make a difference to daily working lives or make things run more smoothly such as reduced waste or duplication.
Process	Factor 2: Credibility of the benefits	Whether benefits to patients, staff and the organisation are visible, are believed by staff and can be described clearly.
Process	Factor 3: Adaptability of improved process	Whether changed processes will continue to meet the need of the organisations and can be maintained when an individual or group of people who initiated it are no longer there.
Process	Factor 4: Effectiveness of the system to monitor progress	Whether data are easily available to monitor progress or assess improvement and whether there are systems to communicate this in the organisation.
Staff	Factor 5: Staff involvement and training to sustain the process	Whether staff play a part in the implementation of changes to processes and the extent of training and development of staff to help sustain these changes
Staff	Factor 6: Staff attitudes towards sustaining the change	Whether staff ideas are taken on board, the opportunity they are given to test these ideas and their belief that this is a better way of doing things that should be preserved.
Staff	Factor 7: Senior leadership engagement	Whether credible and respected senior leaders are seen as promoting and investing their own time in changes.
Staff	Factor 8: Clinical leadership engagement	Whether credible and respected clinical leaders are seen as promoting and investing their own time in changes.
Organization	Factor 9: Fit with the organisation's strategic aims and culture	Whether the changes being made are seen as an important contribution to the overall organisational aims.
Organization	Factor 10: Infrastructure for sustainability	Whether staff, facilities, equipment and policies and procedures are adequate to sustain new processes.

The factors are grouped into three domains entitled ‘Process,’ ‘Staff’ and ‘Organization.’ The ‘Process’ domain explores the credibility of the new practice and the extent to which staff believe it will increase efficiency, make jobs easier, and be continued when current staff leave. The ‘Staff’ domain assesses frontline staff awareness of and involvement in organizational changes and the commitment of clinical and organization leaders. The ‘Organization’ domain assesses the new practice’s ‘fit’ with existing organizational culture, strategic aims, and infrastructure (such as staff, facilities, policies, procedures, and communication systems).

**Figure 1:**
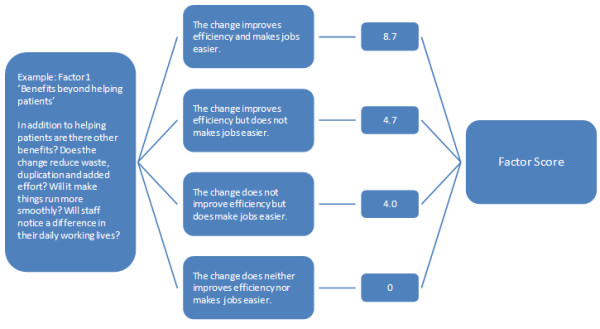
Illustration of scoring mechanism.

For each of the ten factors, individual team members choose from one of four statements or ‘levels’ that they feel represent the ‘best fit’ with their current situation. This is an ordinal scale with the highest level representing the most favorable perspective on sustainability. The model developers used the data from their research (see above) and regression analyses to derive a weighted numerical score for each level of each factor, with the staff domain perceived as most important (52% of total weight), followed by ‘process’ (31%), and ‘organization’ (17%). Figure 1 gives an example of the scoring mechanism for one of the ten factors related to ‘benefits beyond helping patients.’

Individual responses are aggregated into team scores. These team scores, generated at regular intervals as the implementation of new practice progresses, represent the SM’s prediction of the ‘likelihood of sustainability.’ They are intended to raise awareness of determinants of sustainability at an early stage, enable teams to assess their own progress against them, and prompt discussion and action where scores in any of the domains is consistently low.

This article describes an application of the SM by the National Institute for Health Research (NIHR) Collaboration for Leadership in Applied Health Research and Care for Northwest London (CLAHRC NWL), a five-year program supporting frontline care teams implement evidence-based practice using mechanisms such as care bundles, care pathways, reviews and assessments, and new methods of testing for disease. The clinical focus of these projects was varied. Secondary care projects included medicines management (seeking to reduce polypharmacy, improve adherence to prescribing policy, and improve medication administration safety), chronic heart failure, HIV testing at the point of care, and alcohol services. Projects at the interface of primary care and secondary care focused on the implementation of care bundles for patients with community-acquired pneumonia and for patients being discharged from hospital after an acute exacerbation of chronic obstructive pulmonary disease (COPD), as well as projects in primary care or community settings, including vascular risk assessment, sickle cell disease, alcohol use, and improving access to psychological therapies for people with mental health problems. We give our perspective on the potential of the SM to help teams develop and implement new practice and take action likely to improve the prospects of sustainability. Using multiple data points generated from 19 implementation projects over an 18-month period, we explore the ‘acceptability’ or teams’ willingness to engage with the SM and the extent to which it prompts teams to take action to promote sustainability. We discuss the challenges we experienced in applying the SM and briefly outline how we adapted our approach to its use in subsequent cohorts.

### CLAHRC NWL application of the model

CLAHRC NWL supports multidisciplinary teams of approximately 8 to 10 people to implement new evidence-based healthcare practice in their organization. Team membership varied, but typically included a clinical lead (usually a medical consultant or general practitioner (GP)) a project manager, an executive sponsor (usually someone working at or near Board level), and frontline staff delivering care in that disease area. For example, a COPD project team included, in addition to the above, a specialist respiratory nurse, an anti-microbial pharmacist, and a respiratory physiotherapist.

In the early stages of adopting the new practice, teams are given time to plan how to proceed with the intervention, identify what processes may need to change, and who needs to be involved. They then test the impact of changes to care delivery and adapt their approach where necessary using an iterative approach. The SM is used from the beginning alongside a basket of other quality improvement tools, including process mapping, Plan-Do-Study-Act cycles, and real-time measurement as part of an overall methodological approach based on Langley’s Model for Improvement [9].

The rate at which teams adapt new practice to their local care environment will vary, but CLAHRC NWL funds teams for 18 months to develop, test, and implement the required changes to processes of care delivery and secure sufficient engagement and support for new practice in their organization. CLAHRC NWL saw the SM as a potentially useful way to encourage teams to begin building strategies to enhance prospects for sustainability at an early stage of implementation. Distinctions between an initial ‘implementation phase’ and a later ‘sustainability phase’ were seen as unhelpful. Without considering the issues raised by the SM (such as staff involvement, effectiveness of systems to monitor progress, or alignment of new practice with an SM’s organization’s strategic aims), the implementation of new practice may be ineffective. For example, a team may prefer to work in isolation initially to get their new practice ‘right,’ but if they neglect to involve the right people (such as frontline staff or key managers) at an early stage the viability of new practice in that organizational setting may be undermined. Teams are introduced to the SM during initial training sessions and asked to complete it at the start of the project to establish a baseline and subsequently every three months throughout the funded period. Team members anonymously enter their responses using an online reporting tool software system designed by CLAHRC NWL that generates mean overall team scores for each factor and domain and bar charts comparing these against maximum possible scores.

Teams are asked to set aside 5 to 10 minutes in routine meetings to discuss their sustainability scores and to decide what if any actions can be taken to address any factor identified as potential barriers to long-term success. Quarterly completion was chosen to coincide with the quarterly Collaborative Learning and Delivery (CLD) events run by CLAHRC NWL to enable teams to get away from their work environment at regular intervals. Data from the SM are intended to inform team discussions on progress to date and next steps. The scores are also displayed at these events to provide an opportunity to share learning with other teams.

Regular completion of the SM is designed to help capture changing perspectives over time as the challenges to implementation become clear and as team membership changes [10]. Completion by most of the team helps ensure a balance of views across professions, disciplines, and hierarchies. This is important given the variation in SM scores in a previous study that showed frontline clinicians were less optimistic in their scoring than managerial or other support staff [11].

Because aspects of the SM entailed a judgment of sorts on fellow team members (in particular where team members are asked to assess the quality of leadership), anonymous completion of the SM was considered important to enable all team members to express opinions without fear of repercussions.

## Methods

A prerequisite for the successful application of the SM in healthcare settings is the willingness of teams to engage—to complete scores, assess results, and take action where scores indicate a risk of the new practice they are introducing not being sustained. One also needs to have confidence in the validity of the measurement underlying the SM—that the measures used ‘accurately represent the concept being assessed’ [12].

As part of a formative evaluation of the SM to assess the extent to which these prerequisites for successful application appeared to have been met, we triangulated different sets of data available to us as managers of this collaborative program. The teams’ engagement with the SM was assessed using three criteria. The first was the number of sustainability scores registered (assessed through the recording of scores in an online software tool designed by CLAHRC NWL). The second and third criteria are evidence of consideration or discussion of the issues raised by the SM scores and action taken to address concerns on sustainability raised by the SM. For example, if a team’s SM scores indicated concerns over staff involvement (with low scores in the ‘staff’ domain), then evidence of action taken to increase involvement (such as training sessions or staff involvement in the design of new practice) would count towards assessment of engagement.

Evidence of discussion and action relating to sustainability are primarily derived from coding of minutes of ‘two-way’ reviews (where project teams meet with CLAHRC NWL staff for two-hour discussions every six months) and ‘end of project’ reports provided by the teams. Using these data and tacit knowledge of the project acquired through routine regular contact, two CLAHRC NWL staff rate teams independently and compare findings. Differences of opinion were discussed in the wider CLAHRC NWL team until a group consensus was achieved.

To illustrate the types of actions taken in response to SM scores to improve the prospects for sustainability, an example of a project designed to improve the prescribing for the elderly is used. In the early stages of the project, the areas highlighted by SM scores as having the greatest potential for improvement were ‘effectiveness of the system to monitor change’ (in the Process domain) and ‘staff involvement and training’ (in the Staff domain). To improve systems to monitor change, the team worked with CLAHRC NWL to set up a system to regularly measure patients checked for medication they were using, and adverse drug reactions (ADRs), where medications were stopped or the dose was reduced. These data were used by the project team to monitor progress, to feed back to directorates within the hospital to raise the profile of the work, and to illustrate potential cost savings of improved prescribing and reduced ADRs. To improve engagement of frontline staff, the team delivered teaching and training sessions, disseminated awareness-raising material (such as posters and handouts), sent group emails, engaged staff in a Delphi exercise on the design of a tool to record patients’ medication, and ensured key frontline staff were included in the project team. Senior staff were engaged through invitations to join the project team, presentations at meetings regularly attended by managerial and senior clinical staff, and publicizing of the work through hospital communication channels such as newsletters. A Medication Passport, designed with the input of patients, was endorsed by the Royal Pharmaceutical Society and helped to ensure a national profile for the project’s work.

We classified the 19 project teams into three categories—‘engaged,’ ‘partly engaged,’ and ‘not engaged.’ Teams with no entries for one or more quarter were classified as ‘not engaged’ and excluded from subsequent analysis. ‘Engaged’ teams showed evidence of consistent completion of the SM by the majority of team members, discussion of issues raised by the scores, and action taken to promote sustainability. Those with more sporadic quarterly completion and more limited discussion and action taken were classified as ‘partly engaged.’ In a series of charts, we compare the pattern of SM scores for ‘engaged’ and ‘partly engaged’ teams over time and, put simply, whether these make sense based on what we know about action taken by projects to promote sustainability. These data are presented in aggregated form in the article. We also present data in a more granular form in Additional file 1: Appendix A, which shows team-level data, and Additional file 2: Appendix B, which presents scoring trends for all ten key factors.

We hypothesize that if, as claimed, the SM promotes understanding of and measures progress towards sustainability, then over time a closer agreement on the likelihood of the project work being sustained is likely to emerge from the SM scores. To test this hypothesis, we derived a ‘diversity of opinion’ measure to assess variation and the extent to which responses to the model diverge or converge over time. The ‘diversity of opinion’ measure was defined to be the range of the responses for each factor in the model each time a team completed their responses. Thus it is calculated as follows: using the unweighted responses 0 to 3, subtract the lowest response from the highest response given by the specified team on the specified occasion for the specified factor. For each factor a score of 0 indicates no diversity of opinion or full agreement, meaning that every respondent chose the same statement or level of response at that point in time. A score of 3 indicates high diversity, where at least one respondent chose the highest weighted statement and one chose the lowest weighted statement. Scores of 1 or 2 represent intermediate degrees of diversity in the responses. This is a purely descriptive measure, and captures the maximum difference between team members on a given factor, on a given occasion. The range was chosen over other statistical measures of variation (such as interquartile range or standard deviation) because it is appropriate for four-level ordinal scale data and captures the existence of diversity among a small number of measurements, in a simple manner that is straightforward to interpret. It is important to note that this measure is not intended to be associated with any sense of ‘better’ or ‘worse.’ In particular, the authors make no assumption about whether or not a greater diversity of opinion within a team is beneficial or detrimental to success of implementation or to sustainability. At the CLD events referred to above, we also gathered feedback using a simple anonymized questionnaire from team members regarding ease of understanding, ease of application, and usefulness of the SM to explore reasons for variation in engagement.

## Results

Seven of the 19 teams analyzed were categorized as ‘engaged’ , five as ‘partly engaged’ and seven as ‘not engaged’. Tables 2 and 3 show the numbers of staff in ‘engaged’ and ‘partly engaged’ teams completing the SM over time.

**Table 2:** Number of staff completing model per quarter for ‘engaged’ teams

	**Q1**	**Q2**	**Q3**	**Q4**	**Q5**	**Q6**	**Q7**	**Total**
Team 1	8	8	8	8	8	8	8	56
Team 2	15	8	23	2	9	11	9	77
Team 3	8	9	7	9	9	8	8	58
Team 4	10	8	12	6	9	10	8	63
Team 5	8	13	12	8	8	8	10	67
Team 6	8	8	7	9	8	8	8	56
Team 7	9	11	17	9	13	11	8	78

**Table 3:** Number of staff completing model per quarter for ‘partly engaged’ teams

	**Q1**	**Q2**	**Q3**	**Q4**	**Q5**	**Q6**	**Q7**	**Total**
Team 8	8	9	7	8	4	2	4	42
Team 9	13	7	6	2	1	3	3	35
Team 10	8	10	10	6	6	8	5	53
Team 11	7	6	7	10	8	6	6	50
Team 12	5	4	8	1	1	5	3	27

**Figure 2:**
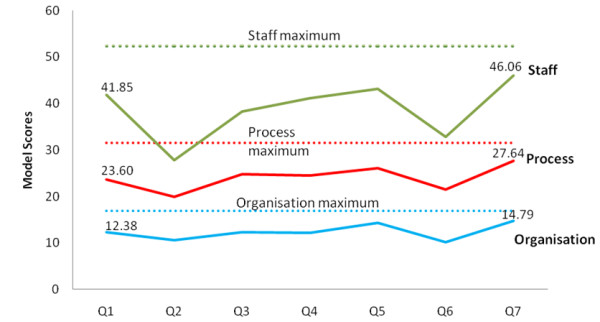
Trends in domain scores for ‘engaged’ teams.

**Figure 3:**
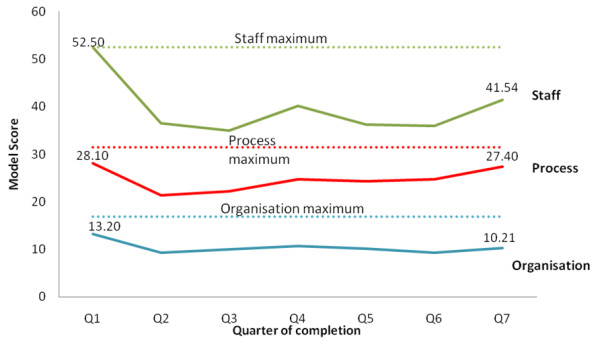
Scoring trends for ‘partly engaged’ teams.

Figures 2 and 3 show aggregated scoring trends for ‘engaged’ and ‘partly engaged’ for the three domains over time. Additional file 1: Appendix A presents the aggregated data of Figures 2 and 3 in a more granular form, and Additional file 2: Appendix B presents data on the scoring trends for each of the 10 factors.

A comparison of ‘engaged’ and ‘partly engaged’ teams shows that scoring trends diverge. Looking at trends over time, Figures 2 and 3 indicates (and Additional file 1: Appendix A confirms) many teams’ scores showing a fall from the initial baseline measure to the second quarter. The first measurement was taken at a preparatory stage of the project. CLAHRC NWL worked with teams in these early stages using methods like process mapping and driver diagrams to plan how to approach implementation in their local organizational environment. It is possible therefore that this fall in the second quarter for many teams represents a tempering of initial optimism or a ‘reality check’ as teams’ understanding of the complexity of the challenges to implementation became clearer.

After this second quarter, the scoring trends of ‘engaged’ and ‘partly engaged’ teams diverge. In the ‘Staff’ domain the scores for ‘engaged’ teams rise between quarters two and five. Chart A1 in Additional file 1: Appendix A shows this is the case for all but one of the teams and Chart B1 in Additional file 2: Appendix B shows this rise is consistent for the four factors in the ‘Staff’ domain. This is consistent with the tendency for these engaged teams to take more action related to the ‘Staff’ domain in this period than ‘partly engaged’ teams. Typical actions to promote sustainability in this domain include: repeated efforts to raise awareness of initiatives beyond staff project team members; gaining the endorsement and support of clinical and managerial staff for their work; identifying through trial and error what group of staff are best placed to assume responsibility for implementing new practice in the long-run and targeting this group for capacity building, and training to promote skills in delivering the improvements; involving staff and patients upon whom successful implementation depends in the design of systems and processes to promote ownership beyond the team; dissemination of work to local, national, and international audiences through conferences and other means to raise the profile and promote the credibility of their work with senior clinical and managerial staff in their own organizations.

Scoring trends for the ‘Process’ domain are also consistent with project activity. As part of the CLAHRC NWL’s program, all teams received training and ongoing support to apply quality improvement methods to experiment with and iteratively adapt their new practice to fit with processes of care delivery. Teams used these to experiment with changes to care delivery processes initially on a small scale, measure impact through Plan-Do-Study-Act cycles and statistical process control, and refine or adapt their approach to implementation where necessary to fit with existing processes of care delivery in their organization. CLAHRC NWL helped teams to define process measures and set up computerized online systems to enable weekly monitoring of progress. A greater tendency for ‘engaged’ teams to focus on process-related issues is consistent with their higher SM scores in this domain. The comparatively higher scores in the ‘Organization’ domain for ‘engaged’ teams also reflect action to raise the profile of their work among senior managers and clinicians and to show how successful implementation of project work can contribute to that organization’s aims.

Interestingly, for many ‘engaged’ teams, particularly in the Staff domain, there is a fall in teams’ scores in the penultimate quarter of measurement The precise reasons for this would need more detailed research, but we suggest this may be another ‘reality check’ as the 18-month project funding nears completion.

It is important to state that it is possible the patterns in Figures 2 and 3 are in fact random. Without a statistical analysis it is not possible to rule this out. However, the scoring trends are in general consistent with activity within the ‘engaged’ teams related to sustainability. Similarly, the lower scores for ‘partly engaged’ teams—teams that completed the SM every quarter, but took little action to promote sustainability—are consistent with what one would expect to see with more limited activity. We present these findings not as definitive proof of validity, but as one source of data that gave us confidence as managers of this program in the validity of the model.

**Figure 4:**
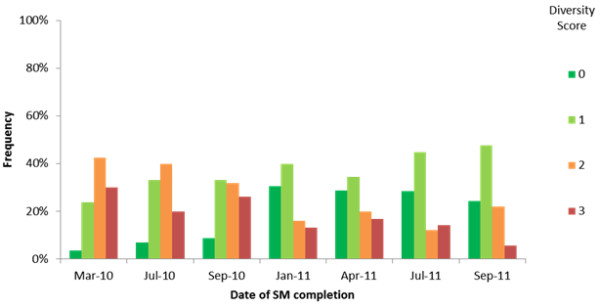
**‘Diversity of opinion’ analysis.** At each point in time, the distribution shows the frequency of occurrence of each diversity score from 0 (full agreement) to 3 (maximum diversity of opinion). Distributions that are positively skewed (higher frequencies in low scores) indicate less diversity of opinion than negatively skewed distributions (higher frequencies in high scores).

This confidence was reinforced by the analysis of diversity of opinion. Figure 4 combines results from all 12 ‘engaged’ or ‘partly engaged’ teams projects and shows how the distribution of diversity of opinion changes over time.

As time progresses, there is a shift towards more agreement within project teams, represented in the figure as a shift towards the left in the distribution. For us, this indicates what one would expect to see with a valid model—a growing consensus on the prospects for sustainability (positive or negative) as the funding period draws to a close. This does not mean to suggest that consensus or diversity of opinion is a good or bad thing, simply that a clear pattern of emerging consensus is apparent from this analysis.

**Figure 5:**
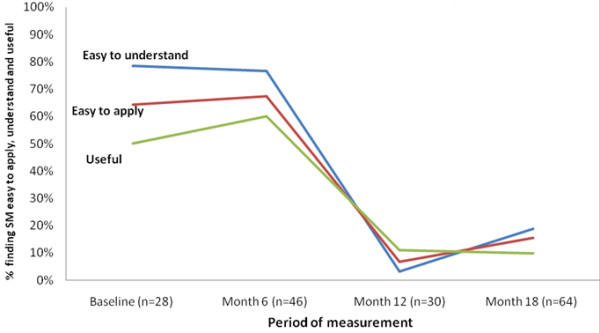
Feedback from team members on SM.

Every six months we asked team members to rate the ease of understanding and application of the model and its usefulness. Figure 5 shows ratings falling considerably as time progresses. Given the challenges we encountered in engaging some teams, negative feedback is not surprising, although the extent shown in Figure 5 was not expected. Because responses are anonymized, it is not possible to assess the proportion of responses in Figure 5 came from ‘engaged,’ ‘partly engaged,’ and ‘non-engaged’ teams. Also, the feedback forms provided very limited information on the reasons for these ratings. However, in the discussion section, we offer some possible explanations based on our ongoing contact and periodic discussions with team members.

## Discussion

As managers of this collaborative program, we aimed to assess strengths and weaknesses of the SM when applied in clinical settings. To the best of our knowledge this is the first study describing the use of the SM by teams to help promote sustainability and it explores, as advocated in a previous study of the SM, ‘whether the SM could be used to diagnose and address sustainability problems in real time’ [10]. It provides a template for how the SM’s effectiveness can be assessed and how it can be used to support teams to focus on factors that affect sustainability.

The data presented here are encouraging in some respects. There are teams who engaged with the SM, have shown an understanding of determinants of sustainability, and taken action to try and improve the prospects of their new practice being sustained. The consistency between SM scores and teams’ propensity to take action to try and promote sustainability and the ‘diversity of opinion’ scores gives us confidence as managers of the program that the SM is measuring what it purports to be.

However, securing engagement is challenging, with seven out of 19 projects not engaged and feedback from teams indicating difficulty in understanding and applying the tool.

The results section showed teams members’ ratings of ease of understanding and application of the SM and its usefulness falling over time. Based on our ongoing contact and periodic discussions with teams, we can highlight common observations of team members that may help explain negative reactions. Some believed that sustainability was an issue to be considered at the end of the project and that there was no value in using the model throughout, especially in the early stages. There were concerns regarding the number of questions and the amount of text in supporting documentation. It was felt by some that the wording of the forced choice statements were inadequate to describe a project’s state of development. For some, terminology used in the model (such as ‘clinical leadership’ or ‘infrastructure’) was difficult to grasp or too vague, and some thought the results were difficult to interpret.

Over time we found that team members tended to fall into one of three camps in terms of attitude: those who had not found a way to engage with the SM at any level and were unenthusiastic; those who were completing it each quarter as required but were sceptical about its value; and those who had engaged and identified ways to make it useful within their projects. In this analysis, levels of engagement with the SM did not appear to be associated with any particular characteristics of the team, setting or type of project.

This study’s analytical approach is designed to assess whether the data from the SM is consistent with our knowledge of what is happening on the ground in projects. There are methodological limitations to this approach, and one needs to be cautious about interpretation. While the data indicate that teams more engaged with the SM took more action to promote sustainability and the level of engagement was positively associated with SM scores, we can not say with certainty that the SM prompted action or that such action would not have occurred in the absence of the SM. Also, because responses were entered anonymously, we did not monitor changes longitudinally by individual team member or team role. Neither was it possible to separate the impact of the SM *per se* from the capacity building used to support it or from the impact of other quality improvement methods designed to help teams make their new practice sustainable. Ultimately, effectiveness depends on whether a project using the SM embeds new working practice as ‘a mainstream way of working within a year or two’ [5], but it is not yet clear whether the actions taken to promote sustainability will achieve this.

Some of these methodological limitations could be addressed using a more traditional research design such as a controlled study. However, much will be learned about how best to apply tools like the SM by repeating studies of this kind, describing how frontline teams react and making use of available data to explore strengths and weaknesses. The testing of initial designs of this or any similar tool in clinical settings will help highlight strengths and weaknesses and aid iterative development.

### Suggestions for future development of SM

Based on what we have learned in our application of the SM so far, we see three areas as important for future effective application of the SM—content, design, and facilitation.

### Content

Many determinants of sustainability proposed in the SM are consistent with those identified in other studies. For example, Shediac-Rizkallah and Bone [13] emphasize similar variables such as: whether a new practice is supported by evidence for its effectiveness; the fit between the intervention and the host organization’s mission and operating routines; the presence of an internal champion to advocate for the program; leadership and whether the program’s key staff or clients believe it to be beneficial; compatibility with an organization’s mission, capacities, and operating procedures’ and risks posed by staff turnover. It is also consistent with a conceptual framework by Edwards *et al*. that emphasizes human and technical resources, opportunities for staff participation, and the alignment of new practice with the organization’s strategic goals and its added value [14]. Sadof emphasizes the importance of measurement of patient outcomes to demonstrate the importance of the new practice to the host organization [15].

However, there is scope to expand the variables considered. We agree with Scheirer’s argument for a stronger emphasis on the political and economic environment as the context for long-term sustainability [8]. The application of the SM in the United Kingdom (UK) is taking place at a time of severe fiscal constraint and large-scale reorganization. For example, the projects included in this article took place at the same time as a drive to find efficiency savings (of £20 billion by 2015 nationally) amid economic upheaval in the UK. From a sustainability perspective, attempts to implement practice were compromised through staff turnover, concerns of frontline staff for their jobs, and management time that could benefit the development of new practice being monopolized by work to achieve these efficiencies [16]. In some cases, participants were uncertain of the continued survival of their organizational units. Future versions of the model may need to incorporate questions on the favorability of external political and economic factors such as these. Another determinant of sustainability that we believe should be more prominent in frameworks of this kind is patient and public engagement, which may be just as important as staff involvement for sustainability. The focus on the fit of new practice with the processes and routines of healthcare organizations risks overlooking whether such new practice meets the needs of patients. Aspects of a similar tool designed for public health programs by the Washington University of St Louis could be incorporated into future versions of the model [17]. While this tool covers much of the same ground as the SM (such as staff awareness and engagement, leadership, adaptability of new practice, and the fit of new practice within the organization’s systems), it includes questions areas of enquiry that could help to address some of the shortcomings highlighted here, such as whether new practice ‘exists in a supportive economic environment’ and the extent of community involvement.

### Design

Concerns were expressed by staff over aspects of the SM’s design that should be considered in future work. The SM is an ambitious attempt to get teams to apply complex ideas, many of which will be unfamiliar to frontline staff. It requires respondents to consider four statements covering ten separate factors affecting sustainability, compare their collective scores against maximum possible scores, and consider what action could or needs to be taken. Future work may be needed to simplify the structure to lighten the ‘cognitive load.’ A more user-friendly design and further development of question wording may help reduce some of the resistance. It may be useful to compare the SM’s design with Washington University’s approach, which poses five questions in eight separate domains with a 7-point scale [17].

### Facilitation

Facilitation of the SM is key to enabling teams to better understand the concepts underpinning the model and encourage teams to apply it. It is important to note that the work described in this study was undertaken in the early stage of a five-year program. Since then, the CLAHRC NWL team have gained more experience in communicating key messages and supporting the application of the SM. We developed novel approaches to training and facilitation in using the SM. On realizing that a didactic approach (with a plenary speaker presenting theory) was having limited success, we changed the way we introduced the SM to subsequent cohorts. This included the use of peer exemplars and allowing teams to brainstorm issues from their experience that may affect sustainability and mapping these to the SM to demonstrate its relevance to their experience. To some teams we also offered facilitated discussion within routine meetings. We observed meetings and mapped the subject matter of team discussions to the factors in the SM. We recommended ways to increase uptake and use of the SM including completing the model at the beginning of the meeting, having administrative support enter the responses during the meeting, and having a later slot on the agenda to discuss the results. Teams are also set challenges such as ‘stakeholder mapping’ exercises to help identify key people that need to be engaged to help promote sustained practice. Attempts to implement the SM elsewhere will need to factor this type of facilitation into their strategy.

## Conclusion

Research has increased our knowledge of what facilitates or impedes the sustained application of evidence-based interventions in local organizational settings [8]. The SM is an important attempt to apply this knowledge to improve the success rate of implementation and deliver more consistent improvements in patient care. It does this by enabling healthcare teams implementing new practice to systematically consider key determinants of sustainability in their organizational environment, provide timely data to assess progress, and prompt action to create conditions for sustained practice. Tools like these need to be tested and assessed for effectiveness in clinical settings and refined where necessary to help make progress in this area.

## Competing interests

There are no competing interests in this article.

## Authors’ contributions

CD led the conception, design and drafting of the paper, TW, CH led on analysis and interpretation of data and RM, CM, KP and JS made substantial contributions to conception and design of the paper. All authors read and approved the final manuscript.

## Supplementary Material

Additional file 1: Appendix A Domain scores by team. Click here for file

Additional file 2: Appendix B Scoring trends by the ten key factors. Click here for file
