# Supplementary figures and images for: Making change last: applying the NHS institute for innovation and improvement sustainability model to healthcare improvement
Source: Implement Sci. 2013 Oct 26;8:127. doi: 10.1186/1748-5908-8-127 (PMC3827618; doi:10.1186/1748-5908-8-127)

**Appendix A: Domain scores by team**


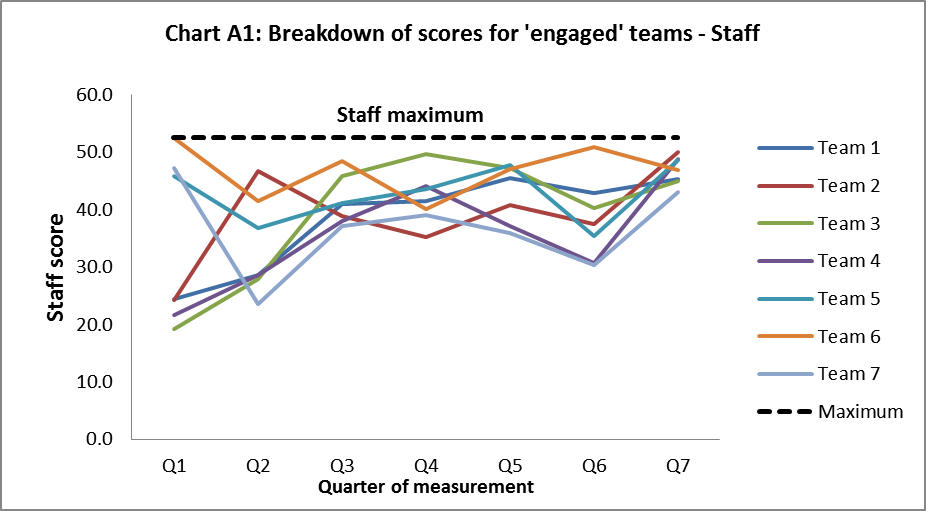


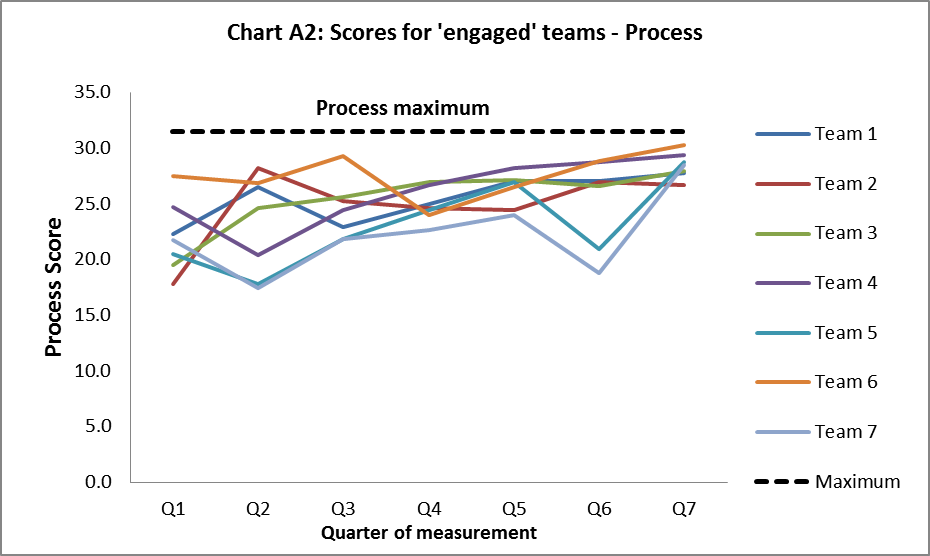


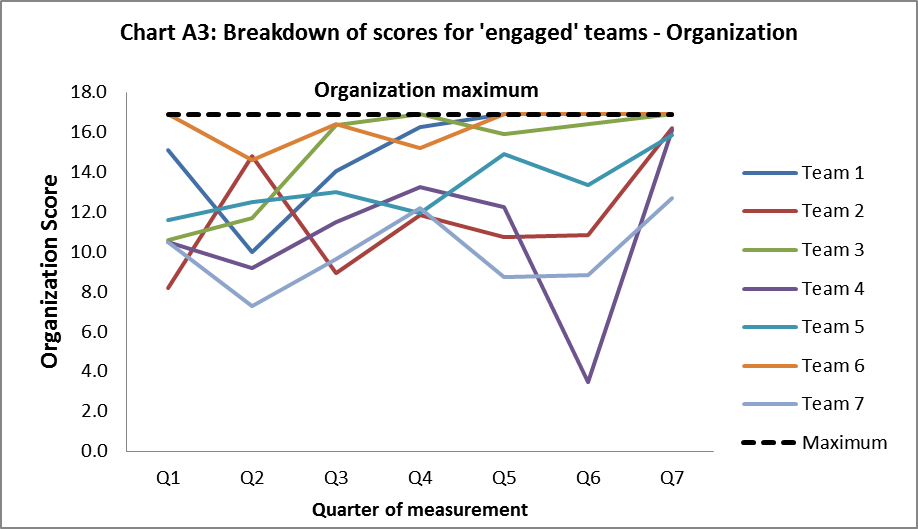


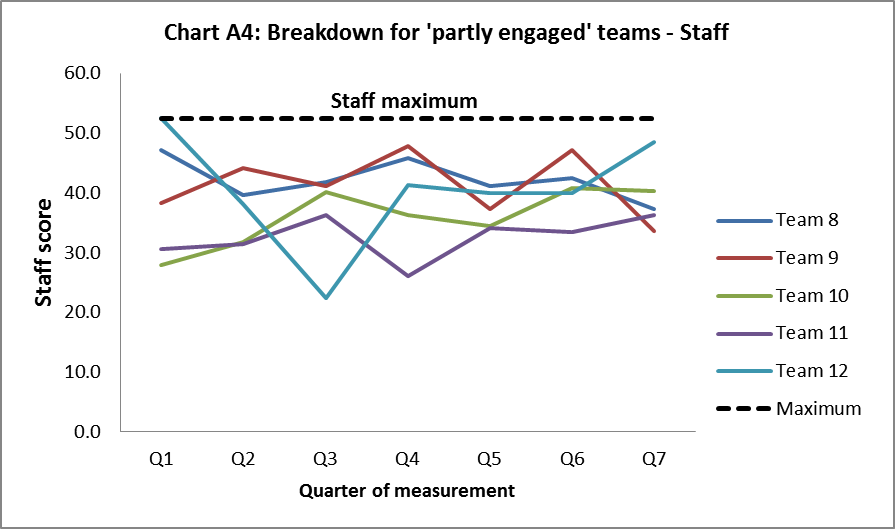


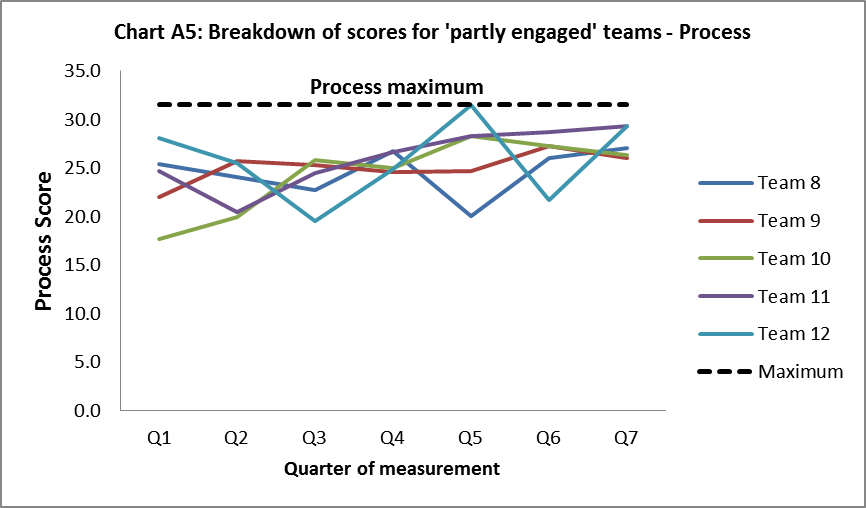


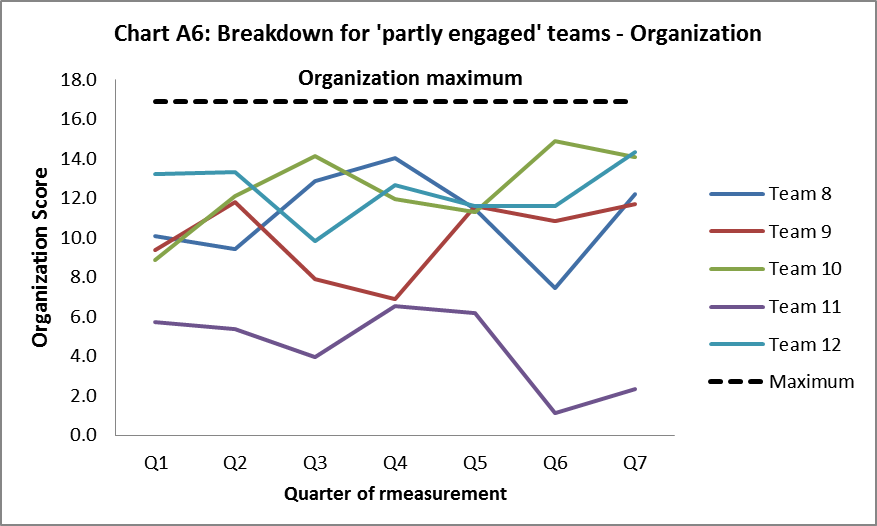

Supplement: Additional file 1: Appendix A — Domain scores by team. [file 1748-5908-8-127-S1.docx]

**Appendix B: Scoring trends by the ten key factors**


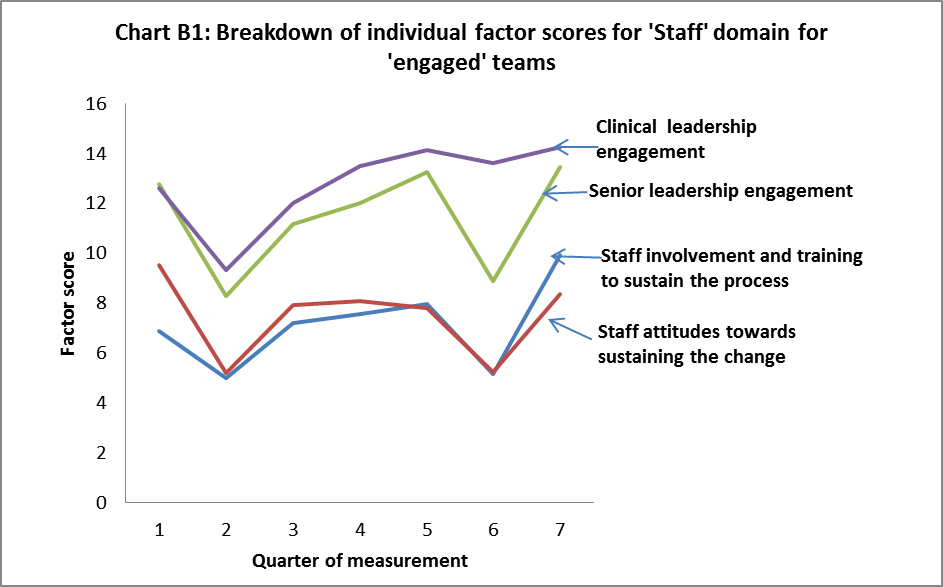


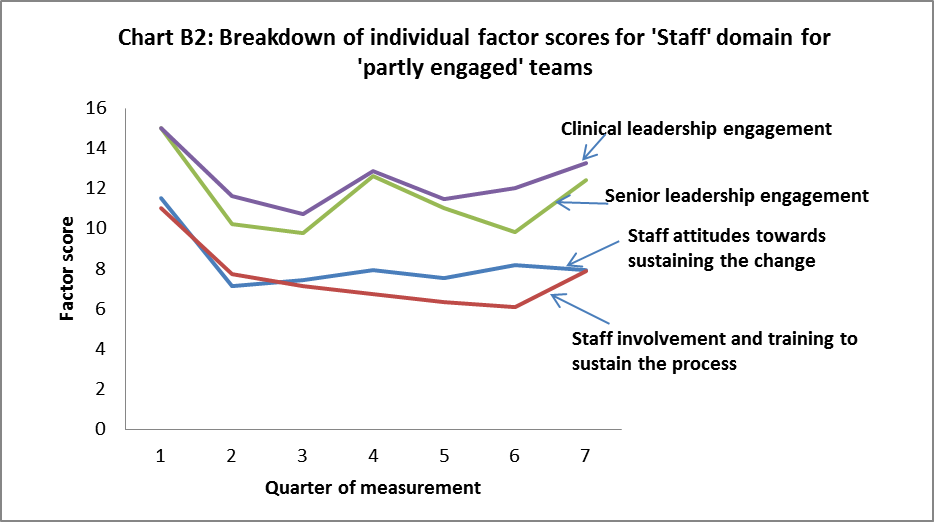


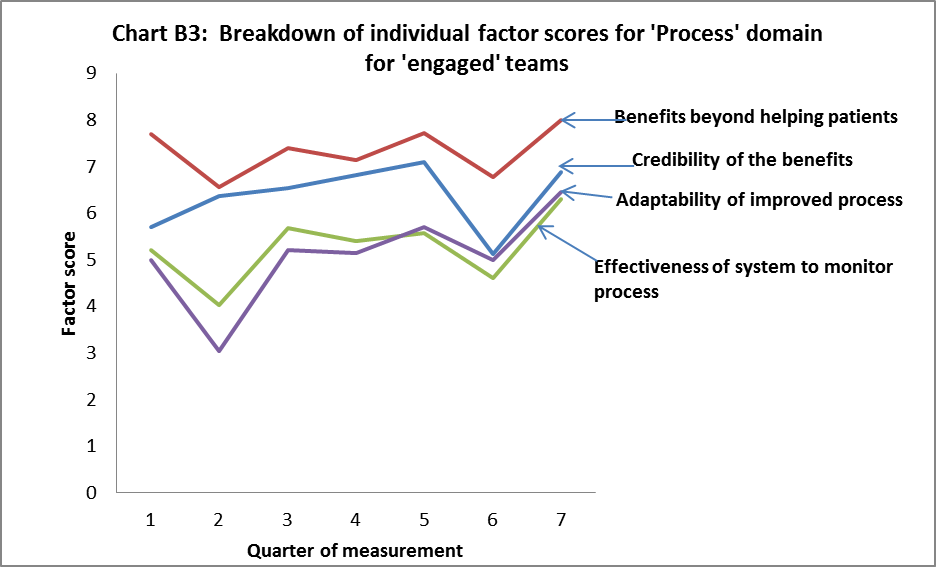


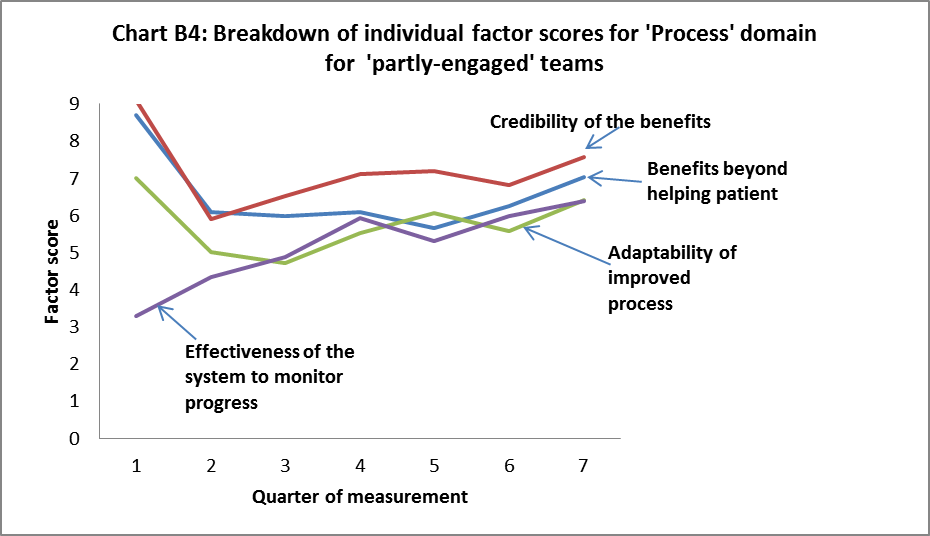


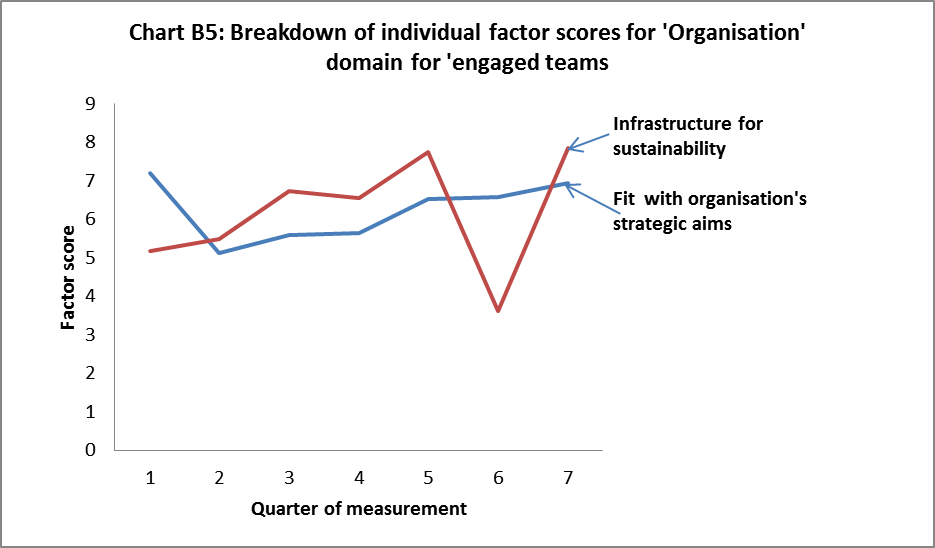


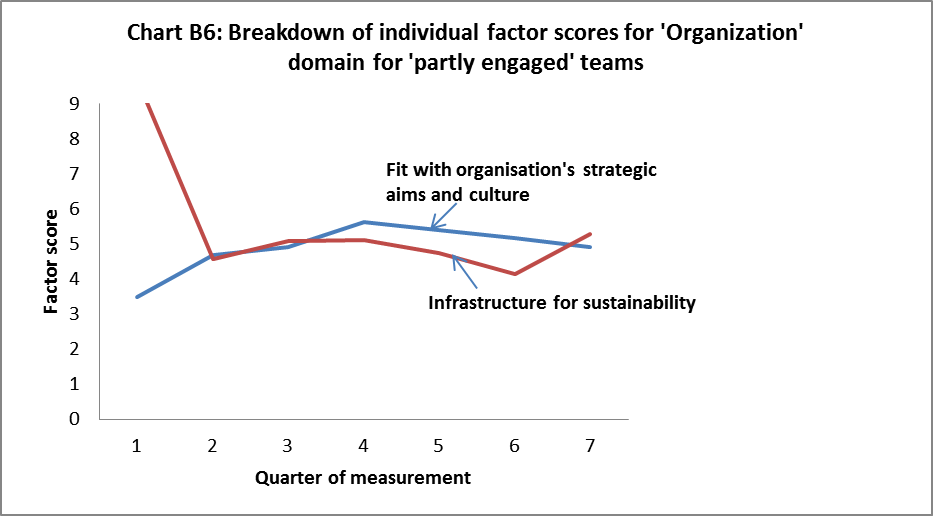

Supplement: Additional file 2: Appendix B — Scoring trends by the ten key factors. [file 1748-5908-8-127-S2.docx]
